# Gender-Based Analysis of Patients Undergoing Mitral Valve Surgery

**DOI:** 10.3390/jcm14197072

**Published:** 2025-10-07

**Authors:** Shekhar Saha, Sophie Meerfeld, Konstanze Maria Horke, Martina Steinmauer, Ahmad Ali, Gerd Juchem, Sven Peterss, Christian Hagl, Dominik Joskowiak

**Affiliations:** 1 Department of Cardiac Surgery, Ludwig Maximillians University of Munich, 80539 Munich, Germany; 2 Department of Internal Medicine I, Department Clinical Medicine, TUM School of Medicine and Health, Technical University of Munich, 80333 Munich, Germany; 3 University Aortic Centre Munich (LMU), LMU University Hospital, 80336 Munich, Germany; 4 Department of Cardiac Surgery, LMU University Hospital, 80336 Munich, Germany; 5 German Centre for Cardiovascular Research (DZHK), Partner Site Munich Heart Alliance, 81377 Munich, Germany

**Keywords:** mitral valve disease, gender

## Abstract

**Objectives***:* To optimise surgical treatment of mitral valve disease (MVD), a better understanding of gender-based differences is required. In this study, we analyse the gender-based differences among patients undergoing mitral valve surgery. **Methods***:* Between January 2019 and December 2024, 809 consecutive patients were admitted to our centre for surgery for MVD. We analysed the patient characteristics, surgical details, postoperative and short-term outcomes of these patients. **Results***:* Females (31.8%) undergoing mitral valve (MV) surgery were older (*p* < 0.001). Females had a higher rate of atrial fibrillation (*p* < 0.001), Rheumatoid arthritis (RA) (*p* = 0.002) and malignancy (*p* = 0.030). Furthermore, females were more often admitted to the intensive care unit (ICU) preoperatively (*p* = 0.037). Among these patients, 419 patients underwent isolated MV surgery. Furthermore, males underwent minimally invasive MV surgery more often (*p* = 0.004). Females had higher rates of combined MVD (*p* < 0.001) and combined MS (*p* < 0.001). Males had higher rates of severe mitral regurgitation (MR) (*p* = 0.041) and Left Atrium (LA) dilation (*p* = 0.004). Females exhibited higher rates of severe Tricuspid Regurgitation (TR) (*p* = 0.032) and pulmonary hypertension (*p* < 0.001). males had higher rates of posterior mitral leaflet (PML) prolapse (*p* < 0.001) and Flail leaflets (*p* < 0.001). Males underwent mitral valve repair (MVr) more often (*p* = 0.002). Early MACCE were reported in 5.1% of the patients. Freedom from major adverse cardiac and cerebrovascular events (MACCE) was comparable at 1 year and three years (*p* = 0.548). Prognosis and freedom from events were comparable between genders. **Conclusions***:* Mitral valve disease presents differently across genders. There exist fundamental differences in the pathophysiological processes and presentation of mitral valve disease. Mitral valve surgery can be carried out with low mortality and morbidity rates irrespective of gender.

## 1. Introduction

**Figure 1:**
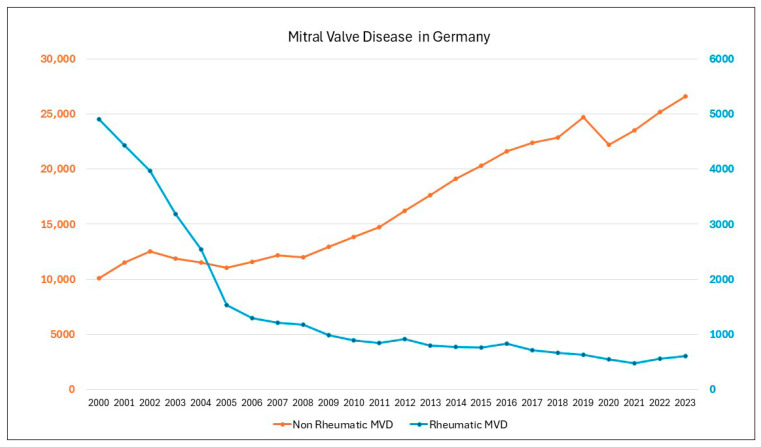
Mitral valve disease in Germany [3].

**Figure 2:**
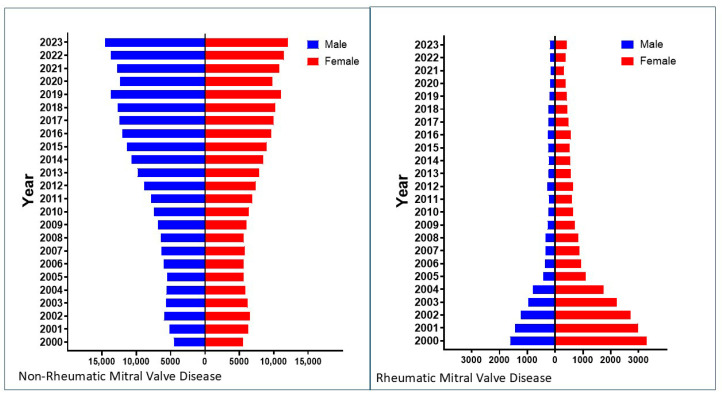
Gender distribution of rheumatic and non-rheumatic mitral valve disease in Germany [3].

Mitral regurgitation (MR) is the most common valvular heart disease worldwide and second most common in Europe, affecting about 1% to 2% of the world’s population [1,2]. In developed countries, mitral valve disease (MVD) accounts for about a quarter of valvular heart disease, with MR being more prevalent than mitral stenosis (MS). In Germany, the number of cases of rheumatic MVD has dropped drastically, whereas the number of cases of non-rheumatic MVD has more than doubled over the last 20 years (Figure 1) [3]. Females accounted for a higher number of cases of rheumatic MVD (Figure 2) [3]. In cases of MVD, gender specific differences are observed at the anatomic and pathophysiologic level [4].

Generally, women suffer from MVD more frequently, whereas males develop aortic valve diseases or aortic stenosis associated with bicuspid aortic valves more often [1]. Females have been reported to have a less-elastic mitral annulus with a larger annular circumference, higher prevalence of rheumatic MVD and atrial secondary mitral regurgitation (ASMR) [4,5]. Furthermore, posterior mitral leaflet (PML) prolapse has been reported to be more common in male patients, whereas anterior mitral leaflet (AML) and bi-leaflet prolapse with myxomatous degeneration have been reported to be more common among female patients [1,4]. To optimise surgical treatment of MVD, a better understanding of gender-based differences is required. In this study, we analyse the gender-based differences among patients undergoing mitral valve (MV) surgery.

## 2. Methods

### 2.1. Ethics Statement

This study was approved by the ethics board of the Ludwig Maximilian University (No. 19-730 and 20-821) and the requirement to obtain patient consent was waived for this retrospective study. Postoperative treatment and data acquisition was performed as part of routine patient care. Data acquisition was based on institutional databases and then de-identified. All procedures described in this study were in accordance with the institutional ethics board and national data safety regulations.

### 2.2. Study Design

We reviewed patients undergoing MV surgery at our center between January 2019 and December 2024. Postoperative treatment and data acquisition was performed as part of routine patient care. All decisions were made in our interdisciplinary Heart-Team. Diagnosis and treatment was determined according to the current ESC/EACTS guidelines [6,7]. We analysed the patient characteristics, individual risk scores, surgical details, postoperative and short-term outcomes of these patients. To predict the postoperative mortality the European System for Cardiac Operative Risk Evaluation II (EuroSCORE II) as proposed by Nashef et al. [8] was calculated. A sub-group analysis was performed to analyse the outcomes following isolated mitral valve surgery. Primary outcomes were 30-day mortality and three-year survival. Major adverse cardiac and cerebrovascular events (MACCE) were defined by mortality of any cause, myocardial infarction and stroke. Early endocarditis was defined as endocarditis occurring within a year of surgery [9].

### 2.3. Data Collection, Statistical Analysis and Illustrations

Data were analysed using IBM SPSS version 29 (Statistical Package for the Social Sciences) (IBM-SPSS Inc., Armonk, NY, USA). Data was tested for normal distribution using the Kolmogorov–Smirnov test with Lillefors correction. Categorical variables were evaluated using the Chi-Squared and Fisher‘s exact method and continuous variables were evaluated using the Mann–Whitney-U test. We used single imputation to replace missing values. Missing continuous values were replaced with the mean value in normally distributed variables and with the median value in non-normally distributed variables. Missing categorical values were replaced with the mode [10]. Survival analysis was performed with Kaplan–Meier curve and log-rank test. All analyses were two tailed. The null hypothesis was rejected, and significant difference was assumed with *p*-values < 0.05. Data are presented as medians (25th–75th quartiles) or absolute values (percentages) unless otherwise specified. Illustrations were prepared using GraphPad Prism v 10 (GraphPad Software Inc, Boston, CA, USA).

## 3. Results

### 3.1. Patient Population

Between January 2019 and December 2024, 809 consecutive patients were admitted to our centre for MV surgery. Among these patients, 419 patients underwent isolated MV surgery. Demographic characteristics are presented in Table 1. Females undergoing MV surgery were older (*p* < 0.001). Females had higher rates of atrial fibrillation (*p* < 0.001), rheumatoid arthritis (RA) (*p* = 0.002) and malignancy (*p* = 0.030). Furthermore, females were more often admitted to the intensive care unit (ICU) preoperatively (*p* = 0.037). Males were treated more often with Single Antiplatelet Therapy (SAPT) (*p* = 0.027) and Vitamin K antagonists (VKA) were used more often in females (*p* = 0.005). There was no difference observed with regard to new oral anticoagulants (NOAC) (*p* = 0.282).

### 3.2. Mitral Valve Pathology and Echocardiographic Data

Preoperative echocardiographic data are depicted in Table 2. Females had higher rates of combined MVD (*p* < 0.001) and combined MS (*p* < 0.001). Males had higher rates of severe MR (*p* = 0.041) and left atrium (LA) dilation (*p* = 0.004). Females exhibited higher rates of severe tricuspid regurgitation (TR) (*p* = 0.032) and pulmonary hypertension (*p* < 0.001). With regard to the MV, males had higher rates of PML prolapse (*p* < 0.001) and Flail leaflets (*p* < 0.001). We observed no differences with regard to AML prolapse and Barlow disease.

### 3.3. Surgical Data

Details of surgery are presented in Table 3. The median cardiopulmonary bypass time (*p* < 0.001) and cross clamping time (*p* < 0.001) was higher among males. Furthermore, males underwent minimally invasive MV surgery more often (*p* = 0.004), and the valve was repaired rather than replaced more frequently (*p* = 0.002) in males than in females We observed no differences with regard to the individual repair techniques. Intra-operative repair failure was reported in 36 patients (4.4%) and was comparable between the groups (*p* = 0.720). Females underwent concomitant tricuspid valve repair more often (*p* < 0.001), whereas males underwent concomitant coronary artery bypass grafting (CABG) procedures more frequently (*p* < 0.001).

### 3.4. Morbidities and Outcomes

Postoperative morbidities and outcomes are listed in Table 4. We observed no differences with regard to postoperative adverse cerebrovascular events, myocardial infarction, new onset atrial fibrillation between the groups.

At discharge, we found no differences regarding the degree of mitral regurgitation between the groups. However, we did observe a higher mean gradient over the mitral valve among females (*p* = 0.002). Furthermore, females had higher rates of mild to moderate tricuspid valve regurgitation (*p* < 0.001). We observed no differences with regard to hospital stay and ICU stay. The total in-hospital mortality of the cohort was 5.1%, and 2.4% among those undergoing isolated MV surgery and was comparable between the groups.

**Figure 3:**
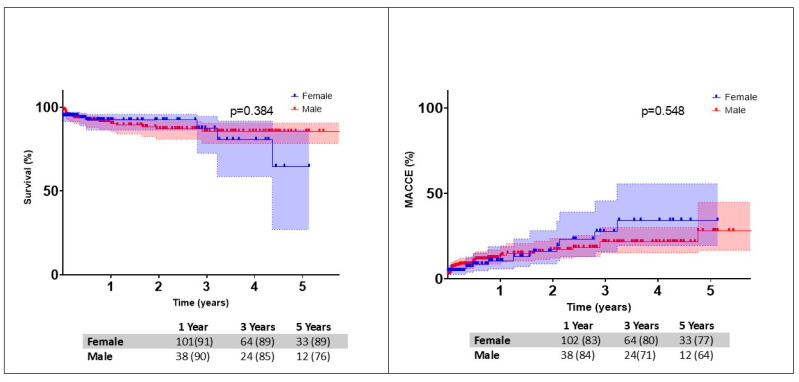
Gender adjusted survival and freedom from MACCE following mitral valve surgery.

On follow-up, a total of 1.5% (n = 12) of patients suffered from adverse cerebrovascular events and 0.1% (n = 1) suffered from myocardial infarctions (Table 5). Early MACCE were reported in 5.1% of the patients. Freedom from MACCE was comparable at 1 year (females 91% vs. males 90%) and three years (females 80% vs. males 71%) (*p* = 0.548). We did not observe any differences between the groups. Survival at 1 year (females 91% vs. males 84%) and 3 years (females 89% vs. males 85%) was comparable between the groups (*p* = 0.384) (Figure 3).

### 3.5. Isolated Mitral Valve Surgery

In our cohort, 419 (51.8%) patients underwent isolated MV surgery. Females undergoing isolated MV surgery were older than males (*p* < 0.001). Females had a higher rate of atrial fibrillation (*p* = 0.014) and had higher rates of NOAC use (*p* = 0.045). Females had higher rates of combined MV disease (*p* = 0.001) and mitral stenosis (*p* = 0.002), whereas males had a higher rate of severe mitral regurgitation (*p* = 0.003) and presented with a PML prolapse more often (*p* = 0.002). Barlow disease was more common in females (*p* = 0.037). Males underwent MVr more frequently than females (*p* = 0.001), whereas females underwent MVR with biological prostheses more frequently (*p* = 0.005). Postoperatively, females had a higher rates of adverse cerebrovascular events (*p* = 0.049) and delirium (*p* = 0.005). The in-hospital mortality was 3.8% among females and 1.7% among males (*p* = 0.299).

## 4. Discussion

### 4.1. Mitral Valve Disease and the Female Patient

MV disease presents differently in males and females. This is due to anatomical and pathophysiological factors [4]. In addition to the above-mentioned differences, male patients may present with more posterior mitral leaflet calcification whereas female patients may present with more annular calcification [1,4]. Females have also been reported to present with generalized myxomatous degeneration of the MV [4]. This is reflected in our cohort too. We found a higher rate of Barlow disease among females undergoing isolated MV surgery.

The 2021 ESC guidelines define mitral regurgitation as severe based on an integrative approach that includes a combination of qualitative, semiquantitative, quantitative, and morphological criteria, such as left ventricular and atrial dilation [2]. As female patients generally have smaller cardiac dimensions than male patients, size-related criteria may contribute to a delayed diagnosis of severe mitral regurgitation in women [4]. This delay may lead to more advanced stages of MVD in females at the time of referral for surgery and could explain the higher prevalence of atrial fibrillation, pulmonary hypertension, and severe tricuspid regurgitation observed in female patients in our cohort. Furthermore, we found that a higher number of females were admitted directly to the ICU. Even among patients undergoing transcatheter MV interventions, women tended to be significantly older [11]. Early referral of females with MVD may lead to improved outcomes.

We found higher rates of TR and pulmonary hypertension among females. TR is associated with poorer outcomes following mitral valve surgery. Atrial fibrillation, rheumatic etiology, dilated atria, LV dysfunction, and preoperative TR have been reported as significant risk factors for TR following MV surgery [12].

In our cohort, females had a higher prevalence of both RA and MS. Typical lesions in RA-associated valvular heart disease include valve nodules and leaflet fibrosis, which may extend to the annulus and subvalvular apparatus [13]. However, MS is more commonly associated with RHD than with RA [14]. Among patients suffering from MVD associated with RA long-term survival after MVr is equivalent to those suffering from RA without MVD. However, patients with RA and MVD have a lower survival as compared to those undergoing surgery without RA [15]. Given that RHD is known to be more prevalent in females, this raises the question of whether RHD may be underdiagnosed in European cohorts with MS [16]. In general, MS and mitral annular calcification have been reported to be more prevalent in females than in men, possibly influencing negative outcomes accordingly [17,18,19].

Another important aspect of MVD is infective endocarditis (IE). In cases of IE, males have been reported to be older and to suffer from aortic valve endocarditis more often, whereas females tend to be younger and suffer from mitral valve endocarditis more frequently [20,21]. Among patients undergoing surgery for IE, female patients have been reported to present with a higher rate of comorbidities, higher surgical risk profile, and have more frequently experienced postoperative morbidities than male patients [22]. In our cohort, we found no differences regarding the incidence of IE between males and females, even among those undergoing isolated MV surgery. Furthermore, upon follow-up, we found the incidence of IE to be as low as 2.1%, with 1.1% of the patients suffering from early endocarditis, and no differences between the groups.

### 4.2. Treatment and Outcomes of Mitral Valve Disease

MVr is the surgical intervention of choice in patients with mitral regurgitation since it is associated with better survival compared with MVR [2]. As durable surgical repair has been reported to be highest for isolated posterior mitral leaflet prolapse, females may have poorer outcomes due to suboptimal surgical repair due to more complex valvular pathologies [4]. In our cohort too, males underwent MVr more frequently, even in cases of isolated MV surgery. Several studies report poorer long term outcomes for females and higher durability of repair among males [23,24]. Andari et al. [25] report that females undergoing MV surgery were found to have increased rates of short-term mortality. In our cohort, we did not observe and differences in the short and mid-term mortality among patients undergoing MV surgery. Furthermore, women have been reported to present with post-operative heart failure more frequently. This may be related to later referral and more advanced disease at the time of surgery compared to men [2]. We observed higher rates of adverse cerebrovascular events and postoperative delirium among females undergoing isolated MV surgery.

Seeburger et al. [26] identified differences in surgical strategies for the correction of MR between genders. They report that females underwent MVR more than twice as often than males. Although several factors such as comorbidities and presenting MV pathology may govern such decisions, the rate of MVr needs to be improved in women. Even though MVR can be performed with comparable outcomes in males and females irrespective of age, the outcomes of MVR are poorer compared to those of MVr [24,27]. Since women present with mitral stenosis or combined MV pathologies more often, repair of stenotic MVs especially due to rheumatic pathology, is not easy in many cases and the surgeon may be forced to replace the valve if repair is considered impossible or non-durable [28]. Furthermore, women tend to be referred later for MV surgery [21,29]. This may be due to several factors, such as lack of index echocardiographic parameters, underestimated severity of MVD and a more fulminant course than in males. Moreover, it has been reported that less than 25% of patients with heart valve disease (specifically mitral and tricuspid regurgitation) who meet indications for referral are referred for invasive treatment [30].

### 4.3. Limitations

This is a retrospective single-centre study with the inherent limitation of such an analysis. Patients undiagnosed or treated conservatively were out of the scope of this study. The small number of patients is associated with a low power of statistical analyses. Due to the small sample size a multivariable analysis was not performed. A longer study duration is required, to account for changing trends such as MICS MV surgery.

## 5. Conclusions

Mitral valve disease presents differently across genders. There exist fundamental differences in the pathophysiological processes and presentation of mitral valve disease. This should be taken into consideration while diagnosing these patients especially while planning surgical interventions. Finally, mitral valve surgery can be carried out with low mortality and morbidity rates irrespective of gender.

## Figures and Tables

**Table 1:** Baseline parameters.

	Mitral Valve Surgery			Isolated Mitral Valve Surgery		
	Female (n = 257)	Male (n = 552)	*p*-Value	Female (n = 133)	Male (n = 286)	*p*-Value
***Patient characteristics***						
Age (years)	69 (62–75)	64 (56–72)	**<0.001**	67 (59–75)	60 (55–69)	**<0.001**
BMI (kg/m^2^)	24 (20.8–28.0)	26 (23.6–28.7)	**<0.001**	23.3 (20.2–26.7)	25.5 (23.5–28.4)	**<0.001**
EuroSCORE II (%)	3.2 (1.7–6.5)	2.4 (1.1–5.4)	**<0.001**	1.9 (1.2–3.5)	1.2 (0.8–2.5)	**<0.001**
Previous cardiac surgery (%)	34 (13.2)	83 (15.0)	0.591	15 (11.4)	40 (14.0)	0.535
***Co-morbidities***						
Arterial Hypertension (%)	168 (65.4)	383 (69.4)	0.258	73 (54.9)	172 (60.1)	0.338
Insulin dependent Diabetes Mellitus (%)	33 (12.8)	66 (12.0)	0.730	14 (10.5)	27 (9.4)	0.726
Hyperlipoproteinemia (%)	89 (34.6)	186 (33.7)	0.811	41 (30.8)	74 (25.9)	0.293
Coronary artery Disease (%)	84 (32.7)	214 (38.8)	0.101	25 (18.8)	59 (20.6)	0.696
ICM (%)	2 (0.8)	9 (1.6)	0.517	0 (0.0)	0 (0.0)	-
DCM (%)	4 (1.6)	17 (3.1)	0.243	1 (0.8)	4 (1.4)	1.000
Peripheral artery disease (%)	33 (12.8)	57 (10.3)	0.337	14 (10.5)	20 (7.0)	0.249
Atrial Fibrillation (%)	135 (52.5)	219 (39.7)	**<0.001**	66 (49.6)	104 (36.4)	**0.014**
Pacemaker (%)	22 (8.6)	33 (6.0)	0.179	6 (4.5)	15 (5.2)	0.815
PCI/PTCA (%)	34 (13.2)	59 (10.7)	0.289	10 (7.5)	18 (6.3)	0.676
Chronic Kidney Disease (%)	40 (15.6)	71 (12.9)	0.324	14 (10.5)	18 (6.3)	0.165
Dialysis (%)	9 (3.5)	21 (3.8)	1.000	2 (1.5)	77 (2.4)	0.725
Immunosuppressive Therapy (%)	9 (3.5)	15 (2.7)	0.514	3 (2.3)	5 (1.7)	0.713
Rheumatoid arthritis (%)	17 (6.6)	11 (2.0)	**0.002**	5 (3.8)	3 (1.0)	0.116
Endocarditis (%)	33 (12.8)	95 (17.2)	0.121	19 (14.3)	44 (15.4)	0.883
Malignancy (%)	37 (14.4)	53 (9.6)	**0.030**	16 (12.0)	25 (8.7)	0.294
Radiation (%)	14 (5.4)	17 (3.1)	0.116	4 (3.0)	6 (2.1)	0.732
IV Drug Abuse (%)	2 (0.8)	0 (0.0)	0.101	1 (0.8)	0 (0.0)	0.318
HIV Infection (%)	1 (0.4)	0 (0.0)	0.318	1 (0.8)	0 (0.0)	0.317
COPD (%)	16 (6.2)	27 (4.9)	0.501	8 (6.0)	15 (5.2)	0.818
Stroke (%)	44 (17.1)	67 (12.1)	0.062	23 (17.3)	33 (11.5)	0.123
Direct ICU admission (%)	44 (17.1)	67 (12.1)	**0.037**	12 (9.0)	23 (8.0)	0.709
Preoperative decompensation (%)	39 (15.2)	63 (11.4)	0.140	16 (12.0)	28 (9.8)	0.497
***Anticoagulants***						
SAPT (%)	59 (23.0)	164 (29.8)	**0.027**	20 (15.0)	54 (18.9)	0.409
DAPT (%)	7 (2.7)	11 (2.0)	0.609	2 (1.5)	5 (1.7)	1.000
VKA (%)	34 (13.2)	38 (6.9)	**0.005**	13 (9.8)	16 (5.6)	0.147
NOAC	82 (31.9)	155 (28.1)	0.282	42 (31.6)	66 (23.2)	**0.045**

**Table 2:** Echocardiographic data on admission.

	Mitral Valve Surgery			Isolated Mitral Valve Surgery		
	Female (n = 257)	Male (n = 552)	*p*-Value	Female (n = 133)	Male (n = 286)	*p*-Value
Aortic Stenosis						
Mild to moderate (%)	21 (8.2)	38 (6.9)	0.562	2 (1.5)	4 (1.4)	1.000
Severe (%)	15 (5.8)	27 (4.9)	0.611	0 (0.0)	1 (0.3)	1.000
Aortic Regurgitation						
Mild to moderate (%)	27 (10.5)	52 (9.4)	0.613	4 (3.0)	2 (0.7)	0.084
Severe (%)	5 (1.9)	23 (4.2)	0.147	0 (0.0)	0 (0.0)	-
Combined mitral valve disease	55 (21.4)	52 (9.4)	**<0.001**	21(15.8)	16 (5.6)	**0.001**
Mitral Stenosis	57 (22.2)	57 (10.3)	**<0.001**	22 (16.5)	18 (6.3)	**0.002**
Mild to moderate (%)	34 (13.2)	27 (4.9)	**<0.001**	11 (8.3)	7 (2.4)	**0.009**
Severe	15 (5.8)	12 (2.2)	**0.011**	7 (5.3)	6 (2.1)	0.126
Mitral Regurgitation	255 (99.2)	545 (98.7)	0.727	132 (99.2)	283 (99.0)	1.000
Mild to moderate (%)	105 (40.9)	205 (37.1)	0.314	56 (42.1)	91 (31.8)	**0.026**
Severe (%)	123 (47.9)	307 (55.6)	**0.041**	64 (48.1)	183 (64.0)	**0.003**
Tricuspid Regurgitation						
Mild to moderate (%)	68 (26.5)	90 (16.3)	**<0.001**	17 (12.8)	27 (9.4)	0.308
Severe (%)	19 (7.4)	22 (4.0)	**0.032**	1 (0.8)	1 (0.3)	0.535
LVEF						
>55% (%)	174 (67.7)	386 (69.9)	0.567	106 (79.7)	238 (83.2)	(0.796)
30–55% (%)	75 (29.2)	144 (26.1)	0.395	27 (20.3)	48 (16.8)	0.412
<30% (%)	0 (0.0)	4 (0.7)	0.313	0 (0.0)	0 (0.0)	-
Pulmonary Hypertension (%)	127 (49.4)	186 (33.7)	**<0.001**	56 (42.1)	69 (24.1)	**<0.001**
LV dilation	38 (27.1)	119 (41.5)	**0.004**	14 (22.6)	51 (37.0)	**0.031**
LA dilation	136 (87.2)	271 (82.1)	0.188	62 (84.9)	131 (80.4)	0.468
Posterior Mitral Leaflet prolapse	94 (36.6)	281 (50.9)	**<0.001**	68 (51.1)	191 (66.8)	**0.002**
P1 Segment	52 (20.2)	131 (23.7)	0.280	36 (27.1)	81 (28.3)	0.816
P2 Segment	87 (33.9)	258 (46.7)	**<0.001**	64 (48.1)	179 (62.6)	**0.006**
P3 Segment	56 (21.8)	156 (28.3)	**0.030**	38 (28.6)	102 (35.7)	0.182
Anterior Mitral Leaflet prolapse	43 (16.7)	104 (18.8)	0.494	20 (15.0)	51 (17.8)	0.576
A1 Segment (%)	36 (14.0)	66 (12.0)	0.427	14 (10.5)	31 (10.8)	1.000
A2 Segment (%)	41 (16.0)	81 (14.7)	0.673	18 (13.5)	38 (13.3)	1.000
A3 Segment (%)	37 (14.4)	83 (15.0)	0.915	15 (11.3)	38 (13.3)	0.637
Flail (%)	69 (26.8)	217 (39.3)	**<0.001**	45 (33.8)	139 (48.6)	**0.006**
Barlow Disease (%)	43 (16.7)	74 (13.5)	0.238	32 (24.1)	46 (16.1)	**0.037**

**Table 3:** Details of surgery.

	Mitral Valve Surgery			Isolated Mitral Valve Surgery		
	Female (n = 257)	Male (n = 552)	*p*-Value	Female (n = 133)	Male (n = 286)	*p*-Value
Duration of CPB (%)	137 (107–177)	159 (126–196)	**<0.001**	118 (94–158)	140 (111–172)	**<0.001**
Duration of Aortic X-clamping (%)	91 (71–114)	103 (80–128)	**<0.001**	77 (63–95)	89 (73–110)	**<0.001**
Minimally invasive surgery (%)	28 (10.9)	105 (19.0)	**0.004**	26 (19.5)	101 (35.3)	**<0.001**
Isolated Mitral valve surgery	133 (51.8)	286 (51.8)	1.000	-	-	-
Mitral valve repair (%)	107 (41.6)	294 (53.3)	**0.002**	67 (50.4)	192 (67.1)	**0.001**
Ring annuloplasty (%)	107 (100.0)	290 (98.6)	0.577	63 (47.4)	200 (69.9)	**<0.001**
Alfieri stich (%)	0 (0.0)	2 (0.7)	1.000	0 (0.0)	2 (0.7)	1.000
Triangular resection (%)	1 (0.9)	6 (2.0)	0.680	0 (0.0)	8 (2.8)	**0.046**
Quadrangular resection (%)	2 (1.9)	8 (2.7)	1.000	1 (0.8)	8 (2.8)	0.283
Neochordae (%)	87 (81.3)	240 (81.6)	1.000	65 (48.9)	191 (66.8)	**<0.001**
Cleft closure (%)	42 (39.3)	95 (32.3)	0.234	29 (21.8)	73 (25.5)	0.464
Mitral valve replacement						
Biological prosthesis (%)	106 (70.7)	167 (64.7)	0.274	41 (30.8)	52 (18.2)	**0.005**
Mechanical prosthesis (%)	44 (29.3)	91 (35.2)	0.228	24 (18.0)	42 (14.7)	0.390
MV repair failure (%)	12 (8.0)	24 (9.3)	0.720	6 (4.5)	12 (4.2)	1.000
Aortic valve surgery						
Biological prosthesis (%)	49 (19.1)	85 (15.4)	0.223	0 (0.0)	0 (0.0)	-
Mechanical prosthesis (%)	5 (1.9)	33 (6.0)	**0.012**	0 (0.0)	0 (0.0)	-
Repair (%)	1 (0.4)	4 (0.7)	1.000	0 (0.0)	0 (0.0)	-
Tricuspid valve surgery						
Biological prosthesis (%)	6 (2.3)	7 (1.3)	0.367	0 (0.0)	0 (0.0)	-
Mechanical prosthesis (%)	1 (0.4)	1 (0.2)	0.535	0 (0.0)	0 (0.0)	-
Repair (%)	61 (23.7)	66 (12.0)	**<0.001**	0 (0.0)	0 (0.0)	-
LV-aneurysm resection (%)	1 (0.4)	6 (1.1)	0.441	0 (0.0)	0 (0.0)	-
VSD closure (%)	1 (0.4)	2 (0.4)	1.000	0 (0.0)	0 (0.0)	-
PFO closure (%)	23 (8.9)	67 (12.1)	0.189	11 (8.3)	34 (11.9)	0.311
LAA closure (%)	125 (48.6)	210 (38.0)	**0.005**	58 (43.6)	96 (33.6)	**0.031**
Ablation (%)	66 (25.7)	130 (23.6)	0.538	35 (26.3)	70 (24.5)	0.717
CABG (%)	21 (8.2)	104 (18.8)	**<0.001**	0 (0.0)	0 (0.0)	-
Aortic surgery (%)	6 (2.3)	26 (4.7)	0.123	0 (0.0)	0 (0.0)	-
Patch plasty (%)	29 (11.3)	37 (6.8)	**0.038**	8 (6.0)	11 (3.9)	0.324

**Table 4:** Morbidities and outcomes.

	Mitral Valve Surgery			Isolated Mitral Valve Surgery		
	Female (n = 257)	Male (n = 552)	*p*-Value	Female (n = 133)	Male (n = 286)	*p*-Value
***Morbidities***						
Adverse cerebrovascular events (%)	11 (4.3)	20 (3.6)	0.695	7 (5.3)	5 (1.7)	**0.049**
Delirium	39 (15.2)	62 (11.2)	0.137	22 (16.5)	20 (7.0)	**0.005**
Surgical site reexploration (%)	43 (16.7)	110 (20.0)	0.290	13 (9.8)	43 (15.0)	0.166
New myocardial infarction (%)	1 (0.4)	9 (1.6)	0.183	0 (0.0)	2 (0.7)	1.000
RCX obstruction	1 (0.4)	7 (1.3)	0.447	1 (0.8)	3 (1.0)	1.000
Renal Replacement therapy (%)	30 (11.7)	68 (12.3)	0.908	6 (4.5)	19 (6.6)	0.508
Pneumonia (%)	97 (37.7)	203 (36.8)	0.815	48 (36.1)	88 (30.8)	0.313
Tracheostoma (%)	7 (2.7)	27 (4.9)	0.189	0 (0.0)	5 (1.7)	0.183
ECLS support (%)	17 (6.6)	39 (7.1)	0.883	5 (3.8)	10 (3.5)	1.000
Duration of ECLS support (%)	5 (3–8)	5 (3–7)	0.633	6 (3–9)	6 (5–7)	0.839
Surgical site infection (%)	14 (5.4)	20 (3.6)	0.259	5 (3.8)	9 (3.1)	0.774
Pacemaker implantation (%)	15 (5.8)	41 (7.4)	0.459	2 (1.5)	8 (2.8)	0.514
New atrial fibrillation (%)	58 (22.6)	122 (22.1)	0.928	28 (21.1)	53 (18.5)	0.595
AV Block III	20 (7.8)	59 (10.7)	0.206	6 (4.5)	12 (4.2)	1.000
***Outcomes***						
Mitral Regurgitation at discharge						
Trace (%)	76 (29.6)	175 (31.7)	0.568	45 (33.8)	97 (33.9)	1.000
Mild to moderate (%)	1 (0.4)	5 (0.9)	0.671	0 (0.0)	4 (1.4)	0.312
Severe (%)	0 (0.0)	1 (0.2)	1.000	0 (0.0)	0 (0.0)	-
Mitral valve PGmax	10 (6–15)	9 (6–15)	0.090	7.3 (5.0–12.0)	12.0 (12.0–12.0)	**0.008**
Mitral valve PGmean	4 (3–5)	3 (2–5)	**0.002**	3.7 (3.0–4.5)	3.0 (3.0–6.0)	**0.027**
Tricuspid Regurgitation at discharge						
Trace (%)	130 (50.6)	301 (54.5)	0.325	76 (57.1)	170 (59.4)	0.671
Mild to moderate (%)	22 (8.6)	16 (2.9)	**<0.001**	9 (6.8)	10 (3.5)	0.139
Severe (%)	1 (0.4)	0 (0.0)	0.318	0 (0.0)	0 (0.0)	-
Hospital stay (days)	16 (13–24)	16 (12–24)	0.350	15 (12–21)	14 (11–19)	**0.046**
ICU stay (days)	5 (4–7)	5 (3–7)	0.465	5 (3–6)	4 (3–6)	0.067
Duration of ventilation (hours)	16 (12–24)	17 (12–24)	0.196	14 (12–24)	15 (12–24)	0.487
In-hospital Mortality (%)	15 (5.9)	26 (4.7)	0.494	5 (3.8)	5 (1.7)	0.299

**Table 5:** Outcomes of Follow-up.

	Total (n = 809)	Female (n = 257)	Male (n = 552)	*p*-Value
Stroke (%)	12 (1.5)	4 (1.6)	8 (1.4)	1.000
New myocardial infarction (%)	1 (0.1)	1 (0.4)	0 (0.0)	0.318
Infective endocarditis (%)	17 (2.1)	3 (1.2)	14 (2.5)	0.294
Early infective endocarditis (%)	9 (1.1)	3 (1.1)	6 (1.1)	0.206
Early MACCE (%)	41 (5.1)	13 (5.1)	28 (5.0)	1.000

## Data Availability

The data underlying this study cannot be shared publicly in accordance with national data safety guidelines, to protect the privacy of individuals that included in the study. The data will be shared on reasonable request to the corresponding author.

## Abbreviations

AML	anterior mitral leaflet
ASMR	atrial secondary mitral regurgitation
BMI	body mass index
CABG	coronary artery bypass grafting
COPD	chronic obstructive pulmonary disease
CPB	cardiopulmonary bypass
DAPT	Dual anti-platelet treatment
DCM	dilative cardiomyopathy
ESC	European society of Cardiology
EuroSCORE II	European System for Cardiac Operative Risk Evaluation II
FFP	fresh frozen plasma
HIV	human immunodeficiency viruses
ICM	ischemic cardiomyopathy
ICU	intensive care unit
IE	infective endocarditis
IV	intravenous
LA	left atrium
LAA	left atrial appendage
LV	left ventricle
LVEF	left ventricular ejection fraction
MACCE	major adverse cardiac and cerebrovascular events
MR	mitral regurgitation
MS	mitral stenosis
MV	mitral valve
MVD	mitral valve disease
MVr	mitral valve repair
MVR	mitral valve replacement
NOAC	new oral anticoagulants
PCI/PTCA	percutaneous coronary intervention/percutaneous transluminal coronary angioplasty
PFO	patent foramen ovale
PG	pressure gradient
PML	posterior mitral leaflet
PRBC	packed red blood cells
RA	rheumatoid arthritis
RCX	ramus circumflexus
RHD	rheumatic heart disease
SAPT	single anti-platelet treatment
sPAP	systolic pulmonary artery pressure
TR	tricuspid regurgitation
VKA	vitamin K antagonist
VSD	ventricular septum defect
